# Exploiting the anisotropy of anomalous scattering boosts the phasing power of SAD and MAD experiments

**DOI:** 10.1107/S0907444908010202

**Published:** 2008-06-18

**Authors:** Marc Schiltz, Gérard Bricogne

**Affiliations:** a École Polytechnique Fédérale de Lausanne (EPFL), Laboratoire de Cristallographie, CH-1015 Lausanne, Switzerland; b Global Phasing Ltd, Sheraton House, Castle Park, Cambridge CB3 0AX, England

**Keywords:** anisotropy of anomalous scattering, phasing, SAD, MAD, polarized resonant diffraction

## Abstract

The X-ray polarization anisotropy of anomalous scattering in crystals of brominated nucleic acids and selenated proteins is shown to have significant effects on the diffraction data collected at an absorption edge. For conventionally collected single- or multi-wavelength anomalous diffraction data, the main manifestation of the anisotropy of anomalous scattering is the breakage of the equivalence between symmetry-related reflections, inducing intensity differences between them that can be exploited to yield extra phase information in the structure-solution process. A new formalism for describing the anisotropy of anomalous scattering which allows these effects to be incorporated into the general scheme of experimental phasing methods using an extended Harker construction is introduced. This requires a paradigm shift in the data-processing strategy, since the usual separation of the data-merging and phasing steps is abandoned. The data are kept unmerged down to the Harker construction, where the symmetry-breaking is explicitly modelled and refined and becomes a source of supplementary phase information. These ideas have been implemented in the phasing program *SHARP*. Refinements using actual data show that exploitation of the anisotropy of anomalous scattering can deliver substantial extra phasing power compared with conventional approaches using the same raw data. Examples are given that show improvements in the phases which are typically of the same order of magnitude as those obtained in a conventional approach by adding a second-wavelength data set to a SAD experiment. It is argued that such gains, which come essentially for free, *i.e.* without the collection of new data, are highly significant, since radiation damage can frequently preclude the collection of a second-wavelength data set. Finally, further developments in synchrotron instrumentation and in the design of data-collection strategies that could help to maximize these gains are outlined.

## Abstract 2

It is shown that the anisotropy of anomalous scattering (AAS) is a significant and ubiquitous effect in data sets collected at an absorption edge and that its exploitation can substantially enhance the phasing power of single- or multi-wavelength anomalous diffraction. The improvements in the phases are typically of the same order of magnitude as those obtained in a conventional approach by adding a second-wavelength data set to a SAD experiment.

## 1. Introduction

Experimental phasing methods based on anomalous scattering (AS), as currently implemented, describe the phenomenon of AS in terms of the wavelength-dependent anomalous scattering factors *f*′ and *f*′′ for certain heavier atoms. These quantities are scalar-valued and hence describe an isotropic AS behaviour without reference to any notion of directionality. It is known, however, that the phenomenon of AS at or very near absorption edges involves an extra degree of complexity, namely anisotropic behaviour through its dependence on the polarization of the X-ray beam, that has not so far been exploited for phase determination.

It is the purpose of this paper to correct this omission and thus complete the task of fully exploiting one further aspect of AS, its anisotropy, for macromolecular phasing. Our rationale is that the move towards ever smaller crystals and more intense X-ray beams has led to radiation damage, rather than beam-time availability, becoming the main limiting factor in the amount of data that can be collected from a single sample for the purpose of experimental phasing. In these circumstances, it becomes of the utmost importance to be able to derive the maximum amount of phase information from data collected at a smaller number of wavelengths (often one), as one cannot count on being able to collect data at further wavelengths from that sample, even if beam time is not the limiting factor.

We show here that the anisotropy of anomalous scattering (AAS), which has so far been overlooked for phasing purposes, is a significant and ubiquitous effect that can deliver substantially enhanced phasing power from conventionally collected single- or multi-wavelength anomalous diffraction (SAD or MAD) data sets after a suitable treatment for it has been introduced into standard phasing software. We also discuss how this effect could be further exploited by suitable adaptations of current instrumentation and experimental protocols.

To situate the present work in its proper context, we first survey certain basic aspects of the use of anomalous scattering for experimental macromolecular phasing (§[Sec sec2]2). As AAS may be unfamiliar to many readers, we give two simple easily visualisable concrete examples of it: firstly in a small-molecule crystal, where it manifests itself through violations of certain systematic absences (§[Sec sec3]3), and secondly through the related effect of breaking the symmetry equivalence of peaks in an anomalous difference Fourier map for a Br-DNA crystal (§[Sec sec4]4). Both examples illustrate the simple conceptual basis of AAS and its ability to give rise to new phase information by breaking the point-group symmetry of the diffraction pattern of a crystal, in much the same way as isotropic AS breaks the Friedel symmetry. We then present a formal treatment of AAS (§[Sec sec5]5) and show that a small approximation to the full expression of an AAS-modulated intensity allows it to be cast in such a form that these intensity modulations, together with a suitable parametrization of the anomalous-scatterer sub­structure, can be used as extra phasing contributors in an extended Harker construction. These extra contributions arise from unmerged intensity measurements between which the usual symmetry equivalence has been broken by AAS. Technical aspects of the modelling of AAS effects and of the refinement of the corresponding parameters are dealt with in §[Sec sec6]6 and are illustrated on the Br-DNA example of §[Sec sec4]4. We then present two other instances of AS phasing supplemented by AAS effects arising from Se atoms: one from a data set specifically collected to further investigate the symmetry-breaking picture of AAS (§[Sec sec7]7) and another from a totally standard data set collected almost a decade ago, without any special concerns regarding methodology, towards a MAD structure determination (§[Sec sec8]8). In both cases we demonstrate substantial gains in phasing power compared with the conventional treatment of AS from the same data, gains that occur, so to speak, ‘for free’.

We conclude (§[Sec sec9]9) by emphasizing the ubiquitousness of AAS effects in data sets measured near the absorption edges of Br or Se and hence the possibility of revisiting such data sets and deriving further phase information from them. We discuss the reasons why AAS had so far remained inconspicuous, how future experimental strategies can be designed with AAS effects in mind so as to maximize the phase-information gain they can afford and how some judicious instrumental choices at synchrotron beamlines could further increase the usefulness of the AAS phasing signal in such experiments.

## 2. Treatment of AS in macromolecular crystallography: a brief survey

Anomalous scattering methods have achieved tremendous success in the structure determination of proteins and nucleic acids using X-ray crystallography (Ogata, 1998 ▶; Hendrickson, 1999 ▶). These methods exploit the resonance behaviour of certain heavier atoms in the macromolecule when the photon energy of the incident X-rays is in the vicinity of one of their absorption edges (Hendrickson, 1991 ▶). Resonance effects lead to the breakdown of Friedel’s law and the resulting intensity differences provide bimodal indications for the structure-factor phases of acentric reflections in the single-wavelength anomalous diffraction (SAD) method. The wavelength-dependent modulation of the anomalous scattering factors *f*′ and *f*′′ in the vicinity of an absorption edge can be further exploited in multi-wavelength anomalous diffraction (MAD) experiments to resolve the bimodality of single-wavelength phase indications of acentric reflections and complement them with phase indications for centric reflections. In all these procedures, the *f*′ and *f*′′ factors are treated as scalars.

**Figure 1:**
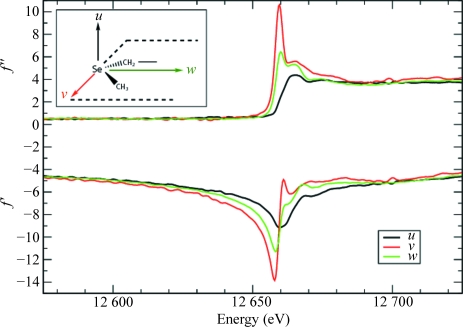
Anomalous scattering factors *f*′ and *f*′′ for Se in selenomethionine residues. The curves represent the anomalous scattering factors when the polarization direction of the incident X-ray beam is aligned with one of the principal molecular directions in a C—Se—C moiety. Black curves: along the direction *u* (perpendicular to the plane containing the C—Se—C bonds). Green curves: along the direction *w* (bisecting the C—Se—C angle). Red curves: along the direction *v* (perpendicular to *u* and *w*). Data from Bricogne *et al.* (2005 ▶).

It has been known since the pioneering work of Templeton & Templeton (1980 ▶) that in the vicinity of an absorption edge AS can display anisotropic behaviour with respect to the polarization of the X-ray beam and is then best described by representing *f*′ and *f*′′ as tensor rather than scalar quantities. This was subsequently confirmed by numerous studies on salt and small-molecule crystals (Templeton & Templeton, 1982 ▶, 1985*a* ▶,*b* ▶, 1986 ▶, 1987 ▶, 1989 ▶, 1991 ▶, 1992 ▶, 1995 ▶; Dmitrienko, 1983 ▶, 1984 ▶; Petcov *et al.*, 1990 ▶; Kirfel *et al.*, 1991 ▶; Kirfel & Petcov, 1992 ▶; Lippmann *et al.*, 1996 ▶; Dmitrienko *et al.*, 2005 ▶). The effects of AAS are most pronounced with (although not restricted to) linearly polarized synchrotron radiation. AAS originates from resonant transitions between the core electrons and antibonding valence molecular orbitals that are rendered nonspherically symmetric by the chemical bonding of the absorbing atom. The anomalous scattering thus depends on the relative orientation of the incident and diffracted electric fields with respect to these molecular orbitals. This is illustrated in Figs. 1 ▶ and 2 ▶ for Se in selenomethionine and Br in brominated nucleotides, respectively, which are the two most important anomalous scatterers used for phasing purposes in macromolecular crystallography.

The relevance of AAS to macromolecular phasing was first pointed out by Templeton & Templeton (1988 ▶) in their study of selenolanthionine, the same small-molecule compound used by Hendrickson (1985 ▶) to demonstrate the possibility of MAD phasing at the Se *K* edge. AAS effects were visible in the dependence of the intensity of X-ray reflections on the orientation of the C—Se—C moieties in the crystal with respect to the polarization direction of the X-ray beam. The variations in anomalous scattering factors produced by changes in crystal orientation were found to be of comparable magnitude to those associated with the changes of wavelength used in the MAD method. Templeton & Templeton (1988 ▶) commented that

the polarization effects reported here may be useful for phasing, but can cause error if not taken into account.

This led Fanchon & Hendrickson (1990 ▶) to investigate the practical impact of AAS on the MAD method. Concerns about the possible detrimental effects of AAS on the accuracy of MAD phasing were alleviated by estimating typical phase errors through numerical simulations and by actual measurements on crystals of selenobiotinyl streptavidin. The authors concluded that

the results show that the AAS does not cripple the MAD method, and that phases uncorrupted by these effects can be recovered.

The potential of AAS to yield extra phase information was essentially left unexplored, although it was pointed out that special scans around the incident-beam direction coupled with a least-squares fitting procedure could yield phase indications. The complicated expression for the full dependence of diffracted intensities on AAS parameters, however, did not seem to be amenable to the incorporation of such effects into any existing framework for phase determination. Following this paper and to this day it became standard practice to overlook the existence of AAS and thus to use exclusively a treatment of AS based on scalar-valued *f*′ and *f*′′.

Our own interest in exploring the potential of AAS for more accurate phase determination, evoked by Templeton & Templeton (1988 ▶), has been longstanding. In an earlier paper (de La Fortelle & Bricogne, 1997 ▶) describing the phasing program *SHARP*, plans to implement a tensorial description for the *f*′ and *f*′′ factors were alluded to. Also, the implementation of refineable ‘anomalous nonisomorphism’ parameters in *SHARP* was in part justified by the fact that AAS effects could be present in SAD or MAD data sets and would give rise to nonisomorphism if the anomalous scattering factors were treated as scalar quantities only. The work described here was preceded by extensive developments of computer code for simulating data affected by AAS and analysing them for the purpose of phase determination (Schiltz & Bricogne, unpublished results). Experimental investigations of AAS effects in macromolecules at the level of polarized absorption spectra were reported by Bricogne *et al.* (2005 ▶) and an example of their impact on the success or failure of a SAD phasing experiment was given by Sanishvili *et al.* (2007 ▶). Here, we finally tackle the central topic of incorporating AAS effects into the general process of macromolecular phasing.

## 3. Anisotropy of anomalous scattering in a nutshell: monoclinic crystals of *p*-bromobenzamide

To illustrate what is involved, we briefly present the example of AAS-induced symmetry-breaking effects in crystals of *p*-­bromobenzamide. In this small-molecule compound, a Br atom is attached to a benzene ring. Thus, the AAS of Br in this compound can be expected to be very similar to that of brominated nucleotides (Sanishvili *et al.*, 2007 ▶). This was confirmed by a series of polarized absorption spectra recorded on triclinic crystals of *p*-bromobenzamide, in which all the C—­Br bonds in the unit cell are parallel (Schiltz *et al.*, unpublished results). As is the case with brominated nucleotides, the white line was found to be most pronounced along the direction parallel to the C—Br bond, whereas it was completely absent if the polarization vector of the X-ray beam was perpendicular to the C—Br bonds. The same molecule can also crystallize in the monoclinic space group *P*2_1_/*c* (unit-cell parameters *a* = 4.491, *b* = 5.415, *c* = 28.013 Å, β = 93.58°) with four symmetry-related molecules of *p*-bromobenzamide in the unit cell. The crystal packing is such that two molecules related by the glide plane have their C—Br bonds in directions that are almost perpendicular to each other (Fig. 3 ▶). X-­ray diffraction experiments were carried out at station BM01A of the Swiss–Norwegian Beamlines (SNBL) at the European Synchrotron Radiation Facility (ESRF) in Grenoble, France. The X-ray beam displays a high degree of linear polarization and the photon energy was tuned to 13.474 keV, which corresponds to the position of the white line at the Br *K* edge.

In an initial experiment, the crystal was oriented so that the polarization direction of the X-ray beam was aligned with the C—Br bond of one of the molecules in the unit cell (Fig. 3 ▶). In this configuration, the C—Br bond of the glide-plane related molecule is almost perpendicular to the polarization direction. Thus, the AAS will cause the two symmetry-related Br atoms to display different anomalous scattering factors at the Br *K* edge: the Br atom that has the C—Br bond aligned with the polarization direction will exhibit a strong white line (large *f*′′), but this will not be the case for its symmetry-related mate, which has its C—Br bond perpendicular to the polarization direction. These Br atoms are therefore no longer equivalent as far as their anomalous scattering properties are concerned and indeed the glide-plane forbidden reflections in the (*h*0*l*) layer are observed to have weak but nonzero intensities (Fig. 3 ▶). It should be mentioned that the diffraction data were collected using a single scan axis which was oriented parallel to the direction of polarization of the X-ray beam. Thus, even though the sample was rotated during data collection, the direction of polarization of the incident beam did not change with respect to the crystal. It was also checked that at energies away from the Br *K* edge the glide-plane forbidden reflections returned to being systematically absent (data not shown).

In a second experiment, the polarization direction of the incident beam was aligned parallel to the glide plane (*i.e.* perpendicular to the crystal *b* axis) and X-ray diffraction data were again collected at the white-line energy position (13.474 keV). This time, the glide-plane forbidden reflections in the (*h*0*l*) layer were observed to have zero intensities (Fig. 3 ▶). The reason for this is easily understood: if the polarization direction of the incident beam is parallel to the glide-plane symmetry element, the C—Br bonds of glide-plane related molecules are oriented at identical angles with respect to the direction of polarization. The equivalence of the symmetry-related Br atoms is thus restored and the forbidden reflections disappear.

This simple example illustrates some of the important aspects of AAS. The electric field vector of the incident X-­ray beam can break the symmetry equivalence between anomalously scattering atoms. As a consequence, the symmetry in reciprocal space is also broken and forbidden reflections can appear. Furthermore, the example shows that the symmetry-breaking effects depend on the relative orientation of the X-­ray polarization direction with respect to the symmetry elements in the crystal. If the polarization direction remains invariant under the action of a symmetry operation of the crystal point group, the corresponding symmetry is preserved, even in the presence of AAS. Below (in §[Sec sec7]7), we will give an example of AAS in a macromolecular crystal where certain symmetries are broken whilst others are preserved owing to the particular orientation of the crystal symmetry elements with respect to the X-ray beam polarization.

## 4. AAS-induced symmetry breaking in crystals of a brominated DNA molecule

The intensity of forbidden reflections stems exclusively from anisotropy in the behaviour of the anomalously scattering atoms. In macromolecules, the proportion of these atoms in the unit cell is usually rather small. As a consequence, forbidden reflections are difficult to observe even if there is significant AAS. On the other hand, the intensity of non­forbidden reflections is a consequence of the superposition of the scattering from both the anomalously scattering atoms and the ‘normally’ scattering atoms in the unit cell. Thus, AAS-induced intensity modulations in nonforbidden reflections can become significant in macromolecular crystals. In the example presented here, we show that AAS by a few Br atoms can give rise to measurable intensity modulations of the nonforbidden reflections in crystals of a brominated DNA molecule.

The brominated Z-DNA duplex d(CGCG[BrU]G) crystallizes in space group *P*2_1_2_1_2_1_, with unit-cell parameters *a* = 17.34, *b* = 32.07, *c* = 44.34 Å. There are two brominated residues per DNA duplex. The packing of the molecules in the crystal is such that all C—Br bonds in the unit cell are located in planes almost perpendicular to [001], but they are not parallel to each other within these planes (see Fig. 4 ▶). We have previously shown (Sanishvili *et al.*, 2007 ▶) that there is a pronounced directional dependence of the anomalous signal strength in X-­ray diffraction data collected at the Br *K* absorption edge from these crystals and that choosing the correct orientation for crystals of such molecules can be a crucial determinant of success or failure when using SAD or MAD methods to solve their structure. Here, we show that beyond the directional dependence of the overall anomalous signal, the anomalous scattering strength of each Br site in the crystal unit cell is modulated by the relative orientation of the corresponding C—Br bond with respect to the X-ray polarization direction.

X-ray diffraction data were recorded from a single cryo-frozen crystal on the SBC-CAT beamline 19ID at the Advanced Photon Source (APS) in Argonne, Illinois, USA (Rosenbaum *et al.*, 2006 ▶) at a wavelength of 0.9199 Å, corresponding to the energy position of the Br *K* edge white line (13.477 keV). The crystal was oriented with the aid of a computer-controlled kappa goniostat and data sets were collected for three different crystal orientations (see Table 1 ▶). The data were recorded using a single scan axis oriented parallel to the direction of polarization of the X-ray beam. Hence, during each data collection the direction of polarization remained constant with respect to the crystal and thus also with respect to the C—Br bonds. Each data set was separately integrated with *MOSFLM* (Leslie, 1993 ▶). Further data processing was carried out with programs from the *CCP*4 software suite (Collaborative Computational Project, Number 4, 1994 ▶). Details of the data collection and reduction are given in Table 1 ▶. Each data set was internally scaled by minimizing the disagreement between symmetry-equivalent reflections in Laue group *mmm*. These scaled intensities were used to produce two reflection files: one with the data merged in the crystal point group 222 and a second with the data merged in point group 1, *i.e.* not imposing any symmetry.

The eight Br sites in the crystal unit cell feature prominently in an anomalous difference Fourier map computed with data merged in the true crystal point group 222. As expected, symmetry-related sites display identical peak heights [see Fig. 5 ▶(*a*) for data set (II)]. However, in anomalous difference Fourier maps computed with the data merged in point group 1, symmetry-related Br sites display widely differing peak heights. Furthermore, for each Br site the peak height also varies considerably among the three data sets, *i.e.* it is a function of crystal orientation. A clear correlation can be established between the height of each peak in the anomalous difference Fourier maps and the angle between the C—Br bond direction and the direction of X-ray polarization for the corresponding Br sites (see Fig. 5 ▶). The anomalous scattering strength is largest for the Br atoms which have their C—Br bonds closely aligned with the X-ray polarization direction. We will analyse this correlation quantitatively in §[Sec sec6.2]6.2 (see also Fig. 6 ▶) and conclude that these large variations in anomalous scattering strengths between symmetry-related Br sites are indeed a manifestation of AAS.

When reflection data are merged in a certain point or Laue group one actually imposes a symmetry on the crystal structure, so that any genuine intensity differences between symmetry-related reflections are averaged out and are instead viewed as contributions to variance estimates. In macromolecular crystallography, data are usually merged in the crystal point group before starting the phase calculation. For this reason, AAS has not up to now been a major obstacle in SAD or MAD phasing in the vast majority of cases. However, if the data are kept unmerged the intensity differences in symmetry-related reflections can be exploited to model the AAS of anomalously scattering atoms. Furthermore, as will be demonstrated below, the AAS-induced intensity differences can yield phase information, which essentially comes ‘for free’. This can be of particular interest for breaking the phase ambiguity in SAD experiments. Another possibility would be to record data at several crystal orientations and to extract phase information from the observed intensity variations. As can be seen from Fig. 5 ▶, each of the three data sets recorded at different crystal orientations on the brominated DNA gives rise to a different configuration of anomalous scatterers in the unit cell (provided that the data are kept unmerged). The situation is analogous to a series of isomorphous crystals in which the ‘anomalous occupancies’ of each individual heavy-atom site would vary from crystal to crystal.

## 5. A formal description of AAS and its connection to macromolecular phasing

Formal derivations that describe the effects of AAS on the intensities of diffracted beams have been reported in the literature in terms of optical models, which were first introduced by Templeton & Templeton (1982 ▶) and further refined by Dmitrienko (1983 ▶), Fanchon & Hendrickson (1990 ▶) and Kirfel *et al.* (1991 ▶). The emphasis in these studies was to describe the intensity modulations of reflections as a function of crystal orientation. Indeed, most experiments that exploit AAS effects in inorganic or small-molecule crystals are carried out by performing azimuthal scans about certain (mainly forbidden) reflections. Thus, the derivations presented, for example, in Kirfel *et al.* (1991 ▶) aim at expressing the diffracted intensity of a reflection as a function of goniometer angles. The experimental measurement of azimuthal scans then allows the extraction of the tensorial properties of anomalous scattering factors.

In macromolecular crystallography the situation is very different, as the vast majority of diffraction experiments use the screenless rotation method with an area detector and with a single scan axis. On most synchrotron beamlines, the scan axis is parallel to the direction of linear polarization of the incident beam (*i.e.* in the plane defined by the orbit of the electron beam and perpendicular to the incident X-ray beam direction). Thus, as the crystal is rotated during data collection, the direction of polarization of the incident beam does not change with respect to the crystal. However, the effects of AAS become apparent through the non-equivalence of symmetry-related reflections. Fanchon & Hendrickson (1990 ▶) have presented a general theory of AAS effects in protein crystallography. However, the general expression for the dependence of diffracted intensities on AAS which they derived (equation 9 in their paper) does not clearly unveil how this phenomenon can be exploited to generate phase information in the standard framework of SAD or MAD phasing. Thus, in their approach phase determination is essentially carried out along conventional lines in the isotropic approximation and the AAS tensors are only refined at the end against unmerged intensities. Here, we show that a considerable simplification in the formalism of AAS can be achieved by considering that those atoms that exhibit AAS properties form only a small subgroup of all the atoms in the unit cell of a macromolecular crystal. This allows us to develop a simple model for the AAS-induced intensity modulations of non­forbidden reflections in macromolecular crystals which can be neatly integrated into the general framework of experimental phasing methods. Using an extended Harker construction, phase information can then be generated through the intensity differences of symmetry-related reflections.

### 5.1. An optical model of AAS in macromolecular crystals

Our model is based on the dipole approximation of anomalous scattering (Finkelstein *et al.*, 1992 ▶; Templeton & Templeton, 1994 ▶; Templeton, 1998 ▶; Ovchinnikova & Dmitrienko, 2000 ▶). The AAS properties of an atom can then be expressed by a second-rank tensor. In macromolecular crystallography, the anomalously scattering atoms are usually in an environment of low symmetry. This is certainly the case for Se in selenomethionine residues and for Br in brominated nucleotides. Thus, the use of the dipole approximation is plainly justified, since higher order effects in AAS are usually substantially weaker and only become visible when the anomalously scattering atoms are in an environment of spherical or cubic symmetry (Finkelstein *et al.*, 1992 ▶; Templeton & Templeton, 1994 ▶). We further assume that the incident X-ray beam is completely linearly polarized. This is a fairly reasonable assumption in the context of macromolecular crystallography since most SAD/MAD experiments are nowadays carried out on undulator beamlines at third-generation synchrotrons, where the degree of linear polarization is usually very high. It is easy to generalize the model presented here to the cases of elliptically and/or partially polarized X-rays, but this will be presented elsewhere.

By convention, all vectors involved in the forthcoming derivations are expressed as column matrices of three components in a crystal Cartesian basis (**e**_*x*_, **e**_*y*_, **e**_*z*_). Thus, our frame of reference is always the crystal, not the laboratory. In the dipole approximation, the X-ray scattering factor of an atom which exhibits AAS is given by a second-rank tensor **f**, which is then represented by a symmetric 3 × 3 matrix with complex-valued entries (Templeton & Templeton, 1982 ▶; Dmitrienko, 1983 ▶), 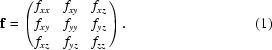
For a given reflection **h**, recorded in a certain geometry, we define a pair of unit vectors, **u** and **v**, that are mutually perpendicular and perpendicular to the incident-beam direction. Similarly, we define a pair of unit vectors, **u**′ and **v**′, that are mutually perpendicular and perpendicular to the scattered-beam direction (Fanchon & Hendrickson, 1990 ▶). The scattering of X-rays from an atom which exhibits AAS is described by a matrix of four elements corresponding to polarization transfers from the incident-beam polarization components **u**, **v** to the scattered-beam polarization components **u**′, **v**′ (Templeton & Templeton, 1982 ▶; Fanchon & Hendrickson, 1990 ▶; Kirfel *et al.*, 1991 ▶),^1^
               
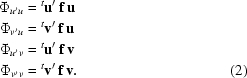
Here, the left superscript *t* denotes matrix transposition.

We now assume that the incident beam is completely linearly polarized along a direction given by the unit vector **p** and we choose the vector **u** to coincide with this direction. Since there is then no polarization component in the incident beam along the direction **v**, only the matrix elements Φ_*u*′*u*_ and Φ_*v*′*u*_ need to be considered. For an atom that displays no AAS, a scattered X-ray beam will also be completely linearly polarized along a direction which we denote by the unit vector **p**′. The direction of **p**′ is obtained by projecting **p** onto a plane perpendicular to the scattered-beam direction. We choose **u**′ = **p**′ and denote **v**′ = **p**′_⊥_. Thus, the two matrix elements that need to be considered are 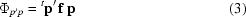
and 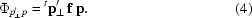
However, for atoms that do not display AAS, only the matrix element Φ_*p*′*p*_ is relevant, since there is no polarization component in the scattered beam along the direction **p**′_⊥_.

The scattering-factor tensor for a given atom can be expressed as a sum of a purely isotropic scattering factor (including isotropic anomalous scattering) and an AAS tensor (Kirfel *et al.*, 1991 ▶), 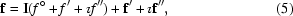
where **I** is the 3 × 3 identity matrix and **f**′ and **f**′′ are the real and imaginary components, respectively, of the scattering-factor tensor that describes the AAS behaviour of that atom. The scattering-matrix elements can then be expressed as 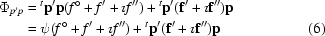
and 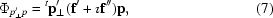
where ψ = ^*t*^ **p**′**p**. It should be noted that ψ^2^ is the standard polarization factor of a reflection when the incident X-ray beam is completely linearly polarized.

Corresponding structure factors are obtained by summing up the scattering contributions of all *N*_uc_ atoms in the crystal unit cell, 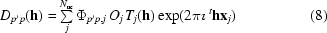
and 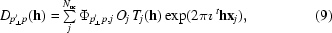
where *O_j_* denotes the occupancy of atom *j*, *T_j_*(**h**) its Debye–Waller factor (temperature factor) and other symbols have their usual meaning.

Using (6) and (7), these summations can be grouped into contributions that are a consequence of purely isotropic scattering on the one hand and of AAS on the other, 
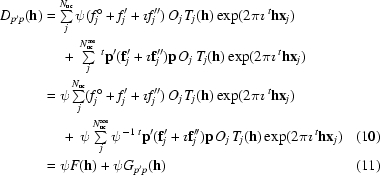
and 
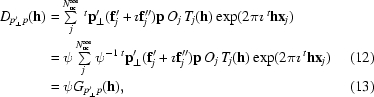
where *N*_uc_^aas^ indicates the number of atoms in the unit cell that exhibit AAS. *F*(**h**) corresponds to the normal isotropic structure factor, including isotropic anomalous scattering.

Since there is no interference between complex contributions with orthogonal polarizations, the intensity of the scattered beam can be expressed as a sum of intensities scattered along the polarization components **p**′ and **p**′_⊥_, 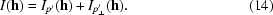
These intensities are related to the squared moduli of the corresponding complex structure factors 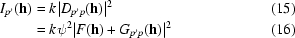
and 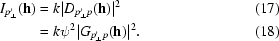
The proportionality factor *k* includes the usual geometric and experimental quantities which relate the integrated intensity of a reflection **h** to the squared modulus of its structure factor, *e.g.* incident-beam intensity, irradiated sample volume, Lorentz correction *etc.*, but excluding the polarization correction, which is explicitly given by the factor ψ^2^.

To summarize, the total intensity can be written as 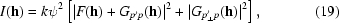
where 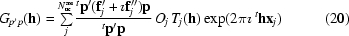
and 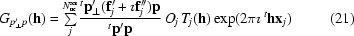
and *F*(**h**) is the normal isotropic scattering factor. In macromolecular crystallography, the proportion of anomalously scattering atoms in the unit cell is usually rather small. The summation in (12) is only over the relatively few atoms which exhibit AAS, whereas the structure factor expressed by (10) is obtained by summing the scattering contributions over all atoms in the unit cell. We can therefore reasonably assume that the second term in (19) is negligible for all but the very weakest reflections and we will use the following approximation for the total intensity of a diffracted beam: 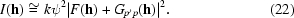
This approximation was also used by Templeton & Templeton (1991 ▶) for the special case of diffraction in the vertical plane to determine phases by polarized dispersion in vanadyl sulfate pentahydrate (*i.e.* using their notation, the *sp*′ scattering factor is ignored).

The physical interpretation of this result is as follows. When the incident X-ray beam is linearly polarized along a direction **p**, the scattering from all the atoms which do not exhibit AAS will give rise to a diffracted beam which is linearly polarized along a direction **p**′. This scattering is expressed by the normal structure factor *F*(**h**). Those atoms which do exhibit AAS will scatter the X-rays in a more complicated way, with resulting polarization components along both the directions **p**′ and **p**′_⊥_. The scattering in each of these polarization components is expressed by the structure factors *G*_*p*′*p*_(**h**) and 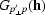, respectively. However, since the scattering in the polarization component **p**′_⊥_ only arises from the relatively few atoms that exhibit AAS, the diffracted intensity in that component, 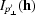, will be a great deal weaker than *I*_*p*′_(**h**). Furthermore, since **p**′ and **p**′_⊥_ are mutually orthogonal, there can be no interference effects between 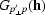 and *F*(**h**). In other words, *G*_*p*′*p*_(**h**) represents that part of AAS which can actually interfere with the scattering from all the other atoms in the unit cell. Experimental phasing methods in macromolecular crystallography are based precisely on interference effects of this kind between the scattering from a so-called substructure, consisting of a relatively small number of atoms (heavy atoms and/or anomalously scattering atoms) that can be modelled and refined, and the scattering from the unknown part of the structure, which consists of the vast majority of atoms. Thus, if the effects of AAS are to be exploited for phase determination, only the contribution *G*_*p*′*p*_(**h**) is of relevance.^2^

### 5.2. Extracting phase information from AAS-induced symmetry-breaking effects

(22) reveals how structure-factor phases for *F*(**h**) can be derived if *G*_*p*′*p*_(**h**) is varied. This equation is formally identical to the equation that defines a circle on the so-called Harker construction (Harker, 1956 ▶), 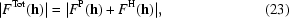
where the total complex structure factor *F* ^Tot^(**h**) is split up into a part *F* ^P^(**h**) that is constant and which arises from a common structure and a variable part *F* ^H^(**h**) which arises from a subset of atoms, often called the substructure. In order to generate phase information, intensity modulations are induced by some physical or chemical changes in the substructure only [*e.g.* a modulation of *F* ^H^(**h**) by wavelength changes in MAD experiments]. It is further required that *F* ^H^(**h**) can be modelled in real space in terms of refineable atomic parameters.

In the presence of AAS, the variable part *F* ^H^(**h**) is given by *G*_*p*′*p*_(**h**). The modulations in *G*_*p*′*p*_(**h**) can be induced by changes in the crystal orientation, *i.e.* by changing **p**. However, even if **p** is fixed, there can be differences in *G*_*p*′*p*_(**h**) and *G*_*p*′*p*_(**h**′), where **h** and **h**′ are symmetry-equivalent reflections.

Even in the absence of AAS, the complex structure factors of symmetry-equivalent reflections are not identical: only their moduli are. Let 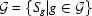 denote the space group of the crystal. The operation of an element *g* of 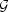 will be written as 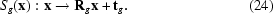
Reflections **h** and **h** *_g_* are symmetry-related^3^ if 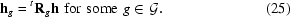
The normal structure factors (*i.e.* the parts which are not affected by AAS) of symmetry-related reflections are related by (Waser, 1955 ▶) 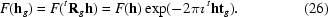
If the symmetry-related reflections *F*(**h** *_g_*) and *F*(**h**) are to be used on the same Harker construction, it is necessary to rotate *F*(**h** *_g_*) back to the phase angle of *F*(**h**), *i.e.* undo the phase shift^4^ exp(−2π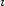 ^*t*^ **ht** *_g_*).

We therefore define 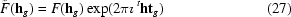
and 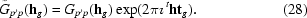
Then, for all 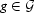, 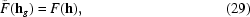
but 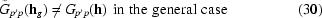
as will be demonstrated in §[Sec sec5.4]5.4.

It can now be seen how AAS-induced phasing can be naturally incorporated into the general framework of *de novo* phasing methods. The measured data are kept unmerged and used as individual contributions (circles) on the Harker construction. Phasing power is generated through the intensity differences between symmetry-related reflections and the associated complex offsets which, according to (30), differ between unmerged observations. In this procedure, the operation that is equivalent to data merging is deferred to the phasing stage. Data merging is effectively replaced by data comparison carried out in the complex plane, *i.e.* through the Harker construction: from all the symmetry-related reflections, a single quantity *F* ^P^(**h**) is estimated, but as a complex value! Evidently, if measured data are available from different crystal orientations, they can also be very naturally incorporated into this scheme.

### 5.3. AAS-induced symmetry breaking in direct space

Using (20), we can write the AAS part of a structure factor as 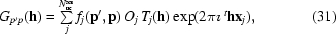
where the AAS contribution of a given atom *j* is noted as 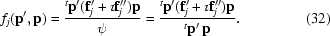
Here, we have made the dependence of *f_j_* on **p**′ and **p** explicit. Using the space-group symmetry, (31) can be rewritten as a summation over *N*_au_^aas^ atoms in an asymmetric unit (assuming that the atoms are not on special positions), 
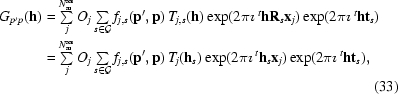
where 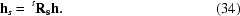
Under the action of the space-group symmetry, the AAS tensors are transformed by a similarity transformation involving the point operators **R** *_s_* (Dmitrienko, 1983 ▶). Thus, 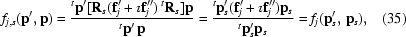
where 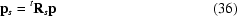
and 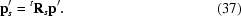
We can now see that the AAS contributions from two atoms that are related by the symmetry operation *s* are identical only if **p**_*s*_ = ±**p** and **p**_*s*_′ = ±**p**′, *i.e.* if both **p** and **p**′ are invariant, up to sign changes, under the operation *^t^* **R** *_s_*.

**Figure 2:**
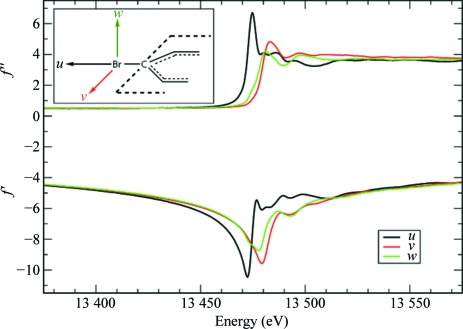
Anomalous scattering factors *f*′ and *f*′′ for Br in brominated nucleotides. The curves represent the anomalous scattering factors when the polarization direction of the incident X-ray beam is aligned with one of the principal molecular directions in a brominated nucleobase. Black curves: along the direction *u* (parallel to the C—Br bond). Red curves: along the direction *v* (perpendicular to the C—Br bond and parallel to the plane of the nucleobase ring). Green curves: along the direction *w* (perpendicular to the nucleobase ring). Data from Sanishvili *et al.* (2007 ▶).

**Figure 3:**
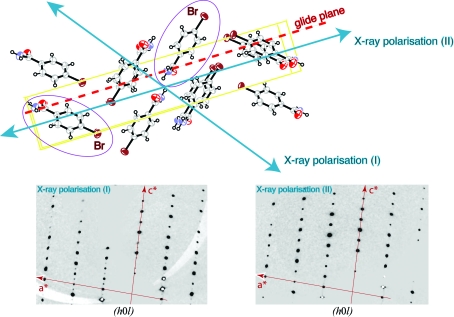
AAS-induced symmetry breaking in *p*-bromobenzamide. The *ORTEP* plot in the upper part of the figure displays the packing of *p*-bromobenzamide molecules in the monoclinic crystal form viewed down the *a* axis. The glide plane is perpendicular to the *b* axis and the translational component is along *c*. Two *p*-bromobenzamide molecules which are related by the glide-plane symmetry operation are highlighted. The direction of linear polarization of the incident X-ray beam is also indicated for two experiments (I) and (II) that were carried out successively. In experiment (I), the C—Br bonds of the two symmetry-related molecules experience the polarization direction at different angles. Thus, in the vicinity of the Br *K* edge, these two Br atoms display different anomalous scattering factors and are no longer equivalent. This symmetry-breaking effect of AAS leads to the appearance of the glide-plane forbidden reflections [(*h*0*l*), *l* = odd] as is shown in the lower left part of the picture, which shows the reconstruction of the (*h*0*l*) layer from experimental data. In experiment (II), the direction of linear polarization of the incident X-ray beam was oriented parallel to the glide plane. The C—Br bonds of the two symmetry-related molecules therefore experienced the polarization direction at identical angles. Thus, in this particular configuration, the symmetry-equivalence of the two Br atoms is restored and the glide-plane forbidden reflections [(*h*0*l*), *l* = odd] are truly absent as is shown in the lower right part of the picture, which shows the reconstruction of the (*h*0*l*) layer from experimental data.

**Figure 4:**
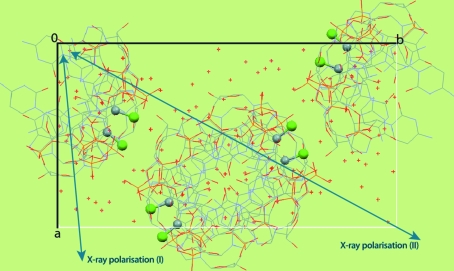
Packing of d(CGCG[BrU]G) molecules viewed down the crystal *c* axis. The eight C—Br moieties in the unit cell are displayed, with the Br atoms highlighted as green spheres. Owing to the orientation of the helical DNA duplexes in the crystal, all C—Br bonds are oriented almost perpendicular to [001]. Also displayed is the in-plane component of the X-ray polarization direction for data sets (I) and (II). {For data set (III), the X-­ray polarization direction was almost parallel to [001] and is therefore not displayed here.}

**Figure 5:**
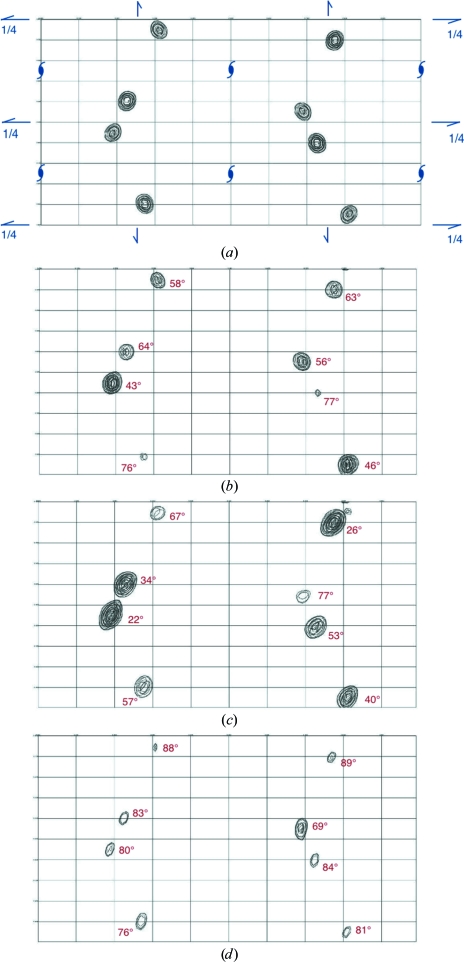
Anomalous difference Fourier maps for d(CGCG[BrU]G) computed to 1.1 Å resolution. The maps are projected down the crystal *c* axis. For each map, the origin is located at the upper left corner and the *a* axis is along the vertical direction. Contours are at intervals of 0.4 e^−^ Å^−3^. (*a*) Map computed from data set (II) merged in point group 222. The symmetry elements of space group *P*2_1_2_1_2_1_ are displayed in blue. (*b*) Map computed from data set (I) merged in point group 1. (*c*) Map computed from data set (II) merged in point group 1. (*d*) Map computed from data set (III) merged in point group 1. The figures printed in red next to each peak indicate the angle between the direction of X-ray polarization and the C—Br bond direction of the corresponding Br site.

As was mentioned above, the direction of **p**′ is obtained by projecting **p** onto the plane that is perpendicular to the scattered-beam direction. At zero scattering angle, **p**′ = **p** and this also holds approximately for small scattering angles.^5^ In macromolecular crystallography, the resolution of the data is usually rather limited and the measurable diffraction is confined to relatively small scattering angles. This is even more true in SAD and MAD experiments that are performed at the *K* edges of Se or Br, which are both located at relatively short wavelengths. Therefore, in many cases, a valid first-order approximation is given by 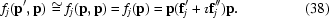
The AAS contribution to a structure factor may then be written as
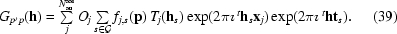
This shows that the dominating effects in AAS-induced intensity modulations in macromolecular crystals arise from the orientation of the incident-beam polarization direction **p** with respect to the arrangements of bonds around anomalously scattering atoms. Thus, in a first-order approximation, the AAS contributions from two atoms that are related by the symmetry operation *s* are identical if **p** is invariant, up to a sign change, under the operation *^t^* **R** *_s_*. In brominated nucleotides, the AAS tensors are almost uniaxial at the white-line energy (see Fig. 2 ▶). Therefore, the AAS of a Br atom is essentially governed by the orientation of **p** with respect to the principal direction of the tensor, *i.e.* the direction of the C—Br bond, as is apparent from the experimental data on brominated DNA presented above.

On synchrotron beamlines, diffraction data for macromolecular crystals are usually recorded with a single scan axis that is oriented parallel to the direction of linear polarization of the incident beam. Thus, as the crystal is rotated during a data collection, the direction of polarization of the incident beam does not change with respect to the crystal so that **p** is the same for all reflections. The factors *f_j_*(**p** *_s_*) are then identical for all reflections and we can drop the dependence on **p** and write 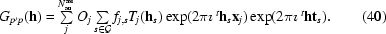
(40) is very similar to the standard expression of the structure factor as a summation over all atoms in the unit cell. The only difference is that for each symmetry-related atom an individual anomalous scattering factor *f*_*j*,*s*_ is defined which depends on *s* ∈ 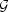 and not only on the label *j* of the symmetry-unique atom. This is the simplest way to model the effects of AAS in macromolecular crystallography. Although this model is strictly speaking only valid at zero scattering angle, we have found it to be a sufficiently good approximation for modelling the major features of AAS in many SAD/MAD data sets from macromolecules. Some examples will be given below (in §§[Sec sec6.2]6.2 and [Sec sec7]7).

### 5.4. AAS-induced symmetry breaking in reciprocal space

Using (35), we can rewrite (33) as 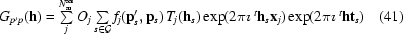
and for a symmetry-related reflection **h** *_g_* 
               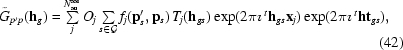
where 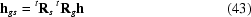
and 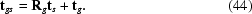
Because of the group properties of 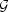, the product *gs* is just another element of 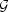, which we denote by *k*. Then, *s* = 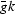, where 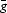 is the inverse of *g*. Thus, 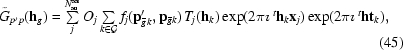
where 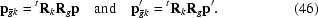
By comparing (41) and (45), it can now be seen that *G*_*p*′*p*_(**h**) and 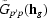 are identical only if 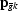 = ±**p** *_k_* and 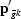 = ±**p**′*_k_, i.e.* if both **p** and **p**′ remain invariant, up to sign changes, under the action of **R** *_g_*.

In the forward-scattering approximation (38), it is enough to state that **p** must be invariant under the action of **R** *_g_* for two reflections **h** and **h**_*g*_ to remain equivalent.

## 6. Modelling and parameter refinement for AAS

The theory presented in the previous section has been implemented as new code in the heavy-atom refinement and phasing program *SHARP* (de La Fortelle & Bricogne, 1997 ▶; Bricogne *et al.*, 2003 ▶). The program had already been extended for the use of unmerged data in the context of radiation-induced phasing (Schiltz *et al.*, 2004 ▶; Schiltz & Bricogne, 2007 ▶). In this new enhanced version, *SHARP* now reads and processes goniometric information in various forms and computes the vectors **p** and **p**′ for each reflection record. By applying the transformations (27) and (28), symmetry-related and/or identical reflections measured at different crystal orientations can be used in the Harker construction, together with data recorded at any other wavelength and/or from any other heavy-atom derivative or native.

The parameters of the heavy atoms are usually first refined in the approximation of isotropic anomalous scattering, *i.e.* with one set of (possibly refineable) *f*′ and *f*′′ parameters per atom or per chemically distinct type of atoms. Once the refinement of the positional and thermal parameters of the anomalously scattering atoms has converged in the isotropic approximation, it is possible to switch on the refinement of AAS parameters. We have implemented two different parametrizations for AAS.

### 6.1. Refinement of AAS tensors

This parametrization implements (33) and (35). For each anomalously scattering atom *j* in the asymmetric unit, the AAS is described by a tensor 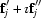, represented by a symmetric 3 × 3 matrix with refineable elements. The contributions of symmetry-related sites are obtained from the transformations (35). The elements of either **f** *_j_*′ or **f** *_j_*′′ or both can be refined. Thus, for each atom in the asymmetric unit, either six or 12 tensorial elements are refined.

This parametrization was tested on the data recorded on the brominated Z-DNA duplex d(CGCG[BrU]G) presented in §[Sec sec4]4. The three data sets corresponding to different crystal orientations were used. For each of the two Br atoms, six individual tensor elements of *f_j_*′′ were refined. Occupancies were fixed to 1 and *f*′ factors were held fixed. No AAS tensors were refined for *f*′, since at the peak wavelength (corresponding to the white line) there is very little anisotropy in *f*′ of Br (see Fig. 2 ▶). The results of the refinement are presented in Table 2 ▶. An eigenvalue/eigenvector analysis yielded the principal directions of the refined tensors. For each Br atom, the principal direction that corresponds to the largest eigenvalue of *f_j_*′′ is in very good agreement with the direction of the C—Br bond (agreement is better than 5.5°), thus validating the refinement. In principle (though probably not in practice) the determination of the tensors could thus be helpful for map interpretation as they give information about the direction of the Se or Br bonding.

The refinement of AAS tensors is very general since it can accommodate data from different crystal orientations. However, if all reflections have been recorded with the same polarization direction **p** (*i.e.* if the crystal is rotated around **p** during data collection, as is almost universally the case in macromolecular synchrotron crystallography) and if the crystal is of low symmetry or if **p** is aligned along a symmetry axis, the refinement of some combinations of tensorial elements may be ill conditioned. This can be seen from (35): if there are only a small number of different **p** *_s_*, only some linear combinations of tensor elements are well defined. In such cases, certain tensor elements or linear combinations of tensor elements can be kept fixed during the refinement.

We have also implemented an alternative description of **f** *_j_*′ and **f** *_j_*′′ tensors in terms of principal values and orientational parameters (Euler angles), as was previously suggested by Fanchon & Hendrickson (1990 ▶). This allows a reduction of the number of parameters since the principal values are identical for all atoms of the same chemical type (at a given wavelength), whereas for a given atom the orientational parameters are identical for different data sets (*e.g.* recorded at different wavelengths).

### 6.2. Refinement of ‘symmetry-unrolled’ anomalous scattering factors

This parametrization implements the forward-scattering approximation (40), where for each anomalously scattering atom in the unit cell (labelled by *j* and *s*) individual anomalous scattering factors *f*_*j*,*s*_′ and *f*_*j*,*s*_′′ are refined. This is only valid if all reflections have been recorded with the same polarization direction **p**. It should be noted that this is not the same as refining all the heavy atoms in space group *P*1. Only the anomalous scattering factors of symmetry-related atoms are refined individually; the positional and thermal parameters are constrained to obey the space-group symmetry, *i.e.* the symmetry-breaking effects are assumed to only originate from the anomalous scattering properties. Also, atoms which are related by lattice-centring translations are constrained to have identical anomalous scattering factors, since the lattice-centring symmetries are not broken by AAS. Either the factors *f*_*j*,*s*_′ or *f*_*j*,*s*_′′ or both can be refined. Thus, for each anomalously scattering atom in the asymmetric unit, either *N*_PG_ or 2 × *N*_PG_ anomalous scattering factors are refined, where *N*_PG_ is the order of the crystal point group. For low-symmetry space groups, this parametrization can therefore be more economical, as far as the number of refined parameters is concerned, than the refinement of AAS tensors. Another advantage of this parametrization is that no goniometric or other information about the crystal orientation is needed. It is therefore the simplest way to implement AAS capabilities in an existing heavy-atom refinement and phasing program. On the other hand, if several data sets have been recorded at different crystal orientations, an individual set of *f*_*j*,*s*_′ and *f*_*j*,*s*_′′ factors must be refined for each data set.

**Figure 6:**
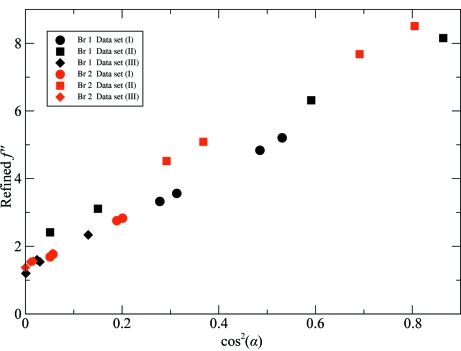
Refined *f*_*j*,*s*_′′ parameters of the Br atoms in the unit cell of d(CGCG[BrU]G) crystals (data from Table 3 ▶) plotted against cos^2^(α), where α is the angle between the C—Br direction and the X-ray polarization direction **p**.

This parametrization was also tested on the data from the brominated DNA. The three data sets were declared as individual batches within the hierarchical organization of data implemented in *SHARP* (de La Fortelle & Bricogne, 1997 ▶). Thus, for each of the two Br atoms, four individual *f*_*j*,*s*_′′ factors (*N*_PG_ = 4 in space group *P*2_1_2_1_2_1_) were refined in each data set. The positional parameters and atomic displacement parameters were constrained to remain identical across all data sets. Occupancies were fixed to 1 and all *f*_*j*,*s*_′ factors were held fixed. The results of the refinement are presented in Table 3 ▶. The refined *f*_*j*,*s*_′′ values correlate very well with the the relative orientation of the C—Br bond with respect to **p** (see Fig. 6 ▶), thus validating the refinement.

### 6.3. Phasing with AAS parameters and unmerged data

**Figure 7:**
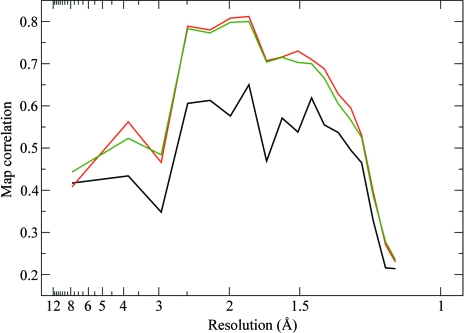
Quality of the phases obtained by exploiting the AAS-induced symmetry-breaking effects in d(CGCG[BrU]G). The plots represent the correlation coefficients, as a function of resolution, of maps computed from experimental phases with a map computed from the final refined structure of d(CGCG[BrU]G). All three data sets (crystal orientations) have been used for phasing. Black, SAD phases computed from merged data with conventional isotropic *f*′′ factors. Red, phases computed from unmerged data using a tensorial description (**f**′′) for the imaginary anomalous scattering factors (see Table 2 ▶). Green, phases computed from unmerged data using distinct *f*′′ factors for ‘symmetry-unrolled’ sites and for each data set (see Table 3 ▶).

For both of the refinements described above (using AAS parameters and unmerged data), phases were computed and compared with the phases obtained from a standard SAD refinement using isotropic *f*′′ factors and merged data. The quality of the phases was assessed through map correlation coefficients (before density modification; presented in Fig. 7 ▶). As can be seen, a substantial improvement of the quality of the phases can be achieved with either of the two alternative AAS parametrizations.

In practical applications, a decision will have to be made at some stage of the refinement on whether the signal is strong enough to support the extra AAS parameters. We are developing a method to compute directional residual maps which can be computed at the end of a standard refinement with isotropic anomalous scattering parameters (Schiltz & Bricogne, unpublished work). These maps will show residual features whenever the data are clearly affected by AAS and will allow an initial estimation of the AAS tensors prior to their refinement.

The modelling and parametrization of non-isomorphism in the case of data affected by AAS is significantly more complex than for standard cases. The error model that is currently implemented in *SHARP* assumes that the effects of all sources of non-isomorphism are uncorrelated between different observations of a given reflection (de La Fortelle & Bricogne, 1997 ▶; Bricogne *et al.*, 2003 ▶). In essence, a diagonal approximation is used for the non-isomorphism covariance matrix. Such an approximation may not always be entirely justified since non-isomorphism can be correlated across observations that have been recorded under similar geometric conditions (Bricogne *et al.*, 2003 ▶). For a more general treatment it will be necessary to resort to multivariate likelihood functions which are capable of accommodating adequate patterns of covariances between the various observations (Bricogne, 2000 ▶; Pannu *et al.*, 2003 ▶). The implementation of these functions in *SHARP* is currently under way.

## 7. AAS and symmetry-breaking effects in crystals of the selenated protein PPAT

The symmetry-breaking effects of AAS were further investigated on crystals of selenomethionine phosphopantetheine adenylyltransferase (PPAT; Izard & Geerlof, 1999 ▶). The motivation behind this study was several-fold. First of all, we aimed at studying the AAS in representative proteins, which are typically much larger molecules than the brominated DNA molecule on which we previously explored AAS. Secondly, the anomalous scatterer investigated here is Se in selenomethionine, which plays a major role in the application of the SAD/MAD methods in protein crystallography as it can be used to replace methionine residues by recombinant DNA technology (Hendrickson *et al.*, 1990 ▶). Polarized dispersion at the Se *K* edge has been observed in crystals of selenomethionine-containing proteins (Hendrickson *et al.*, 1990 ▶; Bricogne *et al.*, 2005 ▶) and related compounds (Templeton & Templeton, 1988 ▶; Fanchon & Hendrickson, 1990 ▶) and revealed that the AAS properties are represented by biaxial tensors (see Fig. 1 ▶). The geometric interpretation of AAS in selenated protein crystals is therefore somewhat more complicated than in brominated DNA or RNA molecules, where the AAS of Br is described to a good approximation by a uniaxial tensor. As a final incentive, the particular protein that was chosen here crystallizes in a cubic space group. It is sometimes thought that the effects of AAS are less pronounced in protein crystals of high symmetry and/or that contain a large number of anomalously scattering atoms in the asymmetric unit because the polarized anomalous scattering from the various atoms would ‘average out’ to isotropy. This is indeed the case for linear dichroism and birefringence, which are global (macroscopic) consequences of AAS and which follow the point-group symmetry of the crystal (Bricogne *et al.*, 2005 ▶; Sanishvili *et al.*, 2007 ▶). Thus, within the validity of the dipole approximation, there can be no dichroism in cubic crystals. However, this is not the case for AAS effects in diffraction, which are microscopic (local) effects to which each individual atom contributes with its own phase shift.^6^

Crystals of PPAT belong to space group *I*23, with unit-cell parameter *a* = 136.23 Å (Izard *et al.*, 1999 ▶). In solution and in the crystal structure, the enzyme forms a homohexamer with point group 32. The threefold axis of the hexamer coincides with the crystallographic ternary axis and its twofold axis gives rise to noncrystallographic symmetry (Izard & Geerlof, 1999 ▶). There are thus two polypeptide chains in the crystal unit cell, each consisting of 159 amino acids and having a molecular weight of 17.8 kg mol^−1^ (kDa). In the selenated form, there are 2 × 9 selenomethionines in the asymmetric unit, but two of them, located in the N-terminal residues, are disordered. The structure of PPAT was solved by three-wavelength MAD phasing at the Se *K* edge (Izard & Geerlof, 1999 ▶).

For the present study, a single crystal of selenomethionine PPAT of approximate dimensions 200 × 200 × 200 µm was mounted on a nylon-fibre loop and flash-frozen in liquid nitrogen. All measurements were carried out on station X25 of the National Synchrotron Light Source (NSLS) at Brook­haven National Laboratory, Upton, New York, USA. This beamline features a three-circle kappa goniometer and is equipped with an ADSC Q315 CCD detector. An absorption spectrum around the Se *K* edge was recorded in fluorescence excitation mode in order to select the wavelength corresponding to the white line. A series of 20 diffraction images were collected in order to compute a crystal orientation matrix. The crystal was then oriented with the aid of the motorized and computer-controlled kappa goniostat so that the [010] direction was aligned with the direction of linear polarization of the incident X-ray beam (**p**). A 240° data set was collected by rotating the crystal about [010]. Thus, the vector **p** remained constantly aligned with the crystal **b** axis. The diffraction images were integrated with *MOSFLM* (Leslie, 1993 ▶). Further data processing was carried out with programs from the *CCP*4 software suite (Collaborative Computational Project, Number 4, 1994 ▶). Details of the data-collection and reduction parameters are given in Table 4 ▶. The data set was internally scaled by minimizing the disagreement between symmetry-related reflections in Laue group *m*3 and two final reflection files were computed, one with the data merged in crystal point group 23 and a second with the data merged in crystal point group 1.

**Figure 8:**
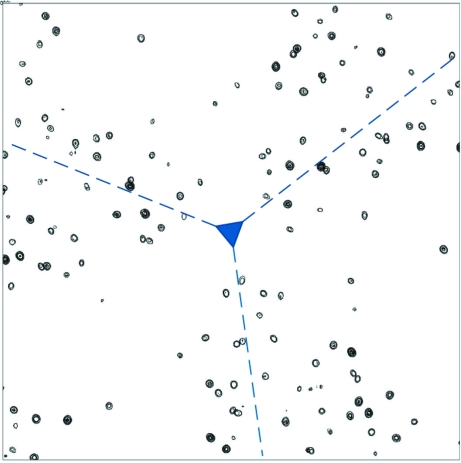
Anomalous difference Fourier map for PPAT computed to 2.2 Å resolution. The map is projected down the [111] axis. The origin is located in the centre of the map and the location of the threefold symmetry axis is displayed in blue. Contours are at intervals of 0.1 e^−^ Å^−3^. The map was computed from the data merged in point group 1. It can be seen that the threefold symmetry is broken.

**Figure 9:**
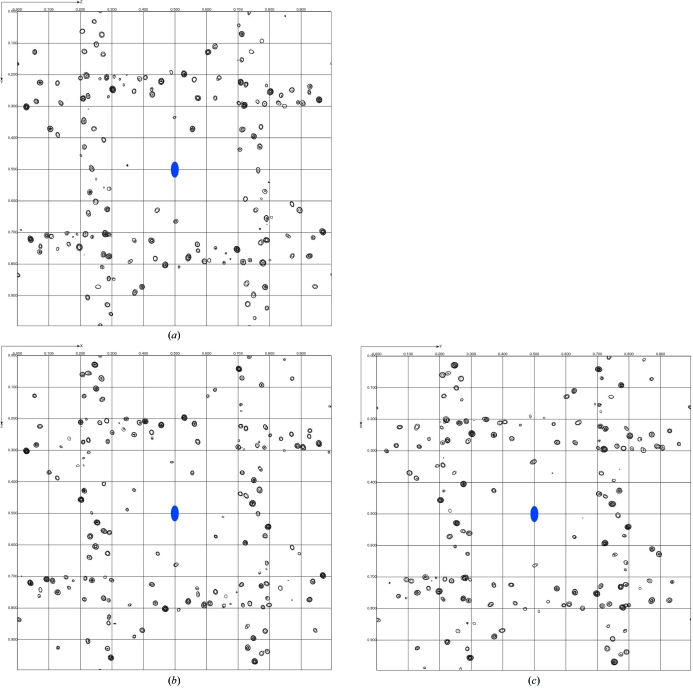
Anomalous difference Fourier maps for PPAT computed to 2.2 Å resolution from the data merged in point group 1. The maps are projected down the axes [100] (*a*), [010] (*b*) and [001] (*c*). For each map, the origin is located at the upper left corner and the location of the twofold symmetry axis is displayed in blue. Contours are at intervals of 0.1 e^−^ Å^−3^.

The Se sites feature prominently in an anomalous difference Fourier map computed with the data merged in the true crystal point group 23. As expected, symmetry-related sites display identical peak heights. However, in an anomalous difference Fourier map computed with the data merged in point group 1, Se sites which are related by a ternary axis display widely differing peak heights (see Fig. 8 ▶). On the other hand, Se sites which are related by a binary axis show similar peak heights (see Fig. 9 ▶).

The parameters of the Se atoms were refined in *SHARP*. Firstly, a standard refinement with isotropic anomalous scattering factors was carried out on the merged data. Coordinates and occupancy parameters were refined for all sites. Based on careful inspection of residual maps, anisotropic displacement parameters were refined for eight Se sites; five additional minor sites, corresponding to alternate positions of main sites, were included in the refinement. Starting from these refined parameters, the unmerged data were used to refine AAS parameters in the form of individual anomalous scattering factors *f*_*j*,*s*_′′ for each Se atom in the unit cell (*i.e.* the parametrization described in §[Sec sec6.2]6.2). No AAS parameters were refined for the minor sites. The final values of the refined AAS parameters are presented in Table 5 ▶. It can be seen that for most symmetry-unique sites, the refined values for the *f*_*j*,*s*_′′ factors fall into three groups. Sites which are related by twofold symmetry operations refine to similar *f*_*j*,*s*_′′ values, whereas this is not the case for sites related by a threefold symmetry operation.

This example nicely illustrates the discussion given in §[Sec sec5.3]5.3. Here, the incident-beam polarization vector **p** was aligned with the [010] direction and thus remains invariant (up to sign changes) under the action of any of the twofold symmetry operations, but not under the action of the threefold symmetry operations along 〈111〉. Thus, to a first-order approximation, the *I*23 symmetry is reduced to *I*222, as is confirmed by the anomalous difference Fourier maps and by the refined values of the AAS parameters.

**Figure 10:**
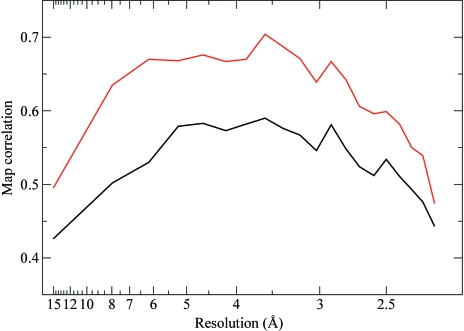
Quality of the phases obtained by exploiting the AAS-induced symmetry-breaking effects in PPAT. The plots represent the correlation coefficients, as a function of resolution, of maps computed from experimental phases with a map computed from the final refined structure of PPAT. Black, SAD phases computed from merged data with conventional isotropic *f*′ and *f*′′ factors. Red, SAD phases computed from unmerged data, using individual *f*′′ factors for symmetry-related sites.

Phases were computed after the refinement of AAS parameters against unmerged data and were compared with the phases obtained from a standard SAD refinement using isotropic *f*′′ factors and merged data (see Fig. 10 ▶). A very significant improvement of the quality of the phases, typically corresponding to a 10–15% increase in map correlation coefficients (before density modification), is achieved by using unmerged data and AAS parameters.

## 8. AAS-induced phasing on ‘standard’ data: improved SAD phasing of the selenated protein IMPDH

As a final example, we present the refinement of AAS parameters and phasing from unmerged data collected at the Se *K* edge on crystals of selenated inosine-5′-monophosphate dehydrogenase (IMPDH; Zhang *et al.*, 1999 ▶). No new data were recorded for this study. Instead, we re-used the data from which the structure was initially solved. Our aim was to assess the improvement in phases that can be achieved on ‘standard’ data (*i.e.* data collected according to conventional strategies), by keeping the data unmerged and by exploiting the AAS effects.

The structure of IMPDH had been solved by three-wavelength MAD phasing from a single crystal of selenated IMPDH (Zhang *et al.*, 1999 ▶). IMPDH crystals belong to space group *I*422, with unit-cell parameters *a* = *b* = 151.49, *c* = 101.67 Å. IMPDH is a homotetramer, with its four subunits related by the crystallographic fourfold axis. The molecular weight of the IMPDH monomer is 52.7 kg mol^−1^ (kDa). It comprises 491 residues, of which 13 are selenomethionines in the selenated form. The MAD data were collected at the SBC-CAT beamline 19ID at the Advanced Photon Source (APS), Argonne, Illinois, USA (Rosenbaum *et al.*, 2006 ▶). At each wavelength, a 90° data set had been recorded to 2.5 Å resolution, using a single scan axis oriented parallel to the direction of polarization of the X-ray beam. The crystal had not been oriented in any special way.

**Figure 11:**
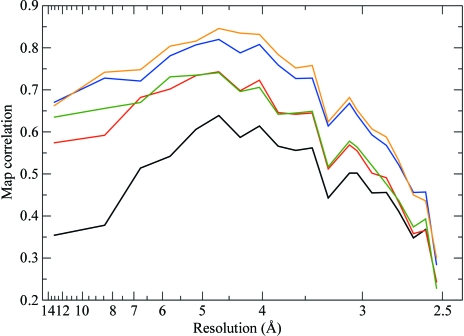
Quality of the phases obtained by exploiting the AAS-induced symmetry-breaking effects in IMPDH. The plots represent the correlation coefficients, as a function of resolution, of maps computed from experimental phases with a map computed from the final refined structure of IMPDH. Black, SAD phases computed from merged data with conventional isotropic *f*′ and *f*′′ factors. Red, SAD phases computed from unmerged data using a tensorial parametrization (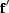 + 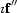) to describe AAS. Green, two-wavelength MAD (peak + inflection point) phases computed from merged data with conventional isotropic *f*′ and *f*′′ factors. Blue, three-wavelength MAD phases computed from merged data with conventional isotropic *f*′ and *f*′′ factors. Orange, three-wavelength MAD phases computed from unmerged data using a tensorial parametrization (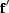 + 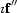) to describe AAS.

In the following, we only used the data set that was recorded at the peak wavelength (λ = 0.9791 Å). Firstly, a standard refinement with isotropic anomalous scattering factors was carried out in *SHARP* on the merged data. Coordinates and occupancy parameters were refined for all sites. Based on the inspection of residual maps, two Se sites were found to have alternate positions and were modelled accordingly. Another Se site showed signs of disorder and was refined with anisotropic displacement parameters. A single *f*′′ parameter was refined and converged to a value of 6.2. The quality of the resulting SAD phases, gauged through map correlation coefficients, is indicated in Fig. 11 ▶.

The raw data were then reprocessed in order to keep the reflections unmerged and goniometric information was extracted for each reflection record. This data set consists of a total of 272 576 observations (for 20 627 unique reflections). Starting from the refined parameters of the Se atoms, AAS tensors (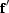 + 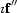) were refined for all sites, along with positional and thermal parameters. The occupancies of all Se atoms were held fixed at their previously refined values. This was necessary in order to avoid instabilities in the refinement which arise from the fact that the occupancy parameters strongly correlate with the diagonal elements of the AAS tensors. The refinement of the elements of **f**′′ proceeded smoothly and the final refined tensors all had eigenvalues of the order of 3, 6, 8.5 (±1.5). The corresponding eigenvectors concur with the principal molecular directions in the various C—Se—C moieties. The refined **f**′ tensors all have eigenvalues which are not widely spread, in the range of −5, −6, −7 (±1.5), thus reflecting the fact that there is relatively little anisotropy in the *f*′ factors at the peak energy of the Se *K* edge (se Fig. 1 ▶).

Phases were computed and their quality in terms of map correlation coefficients is indicated in Fig. 11 ▶. For reference, the quality of the phases obtained by conventional SAD, as well as two- and three-wavelength MAD (on merged data, with isotropic anomalous scattering factors), is shown in Fig. 11 ▶. By using unmerged data and a parametrization for AAS, a very substantial improvement in the quality of the SAD phases is achieved over the standard procedure. This is quite remarkable since no new data were used to obtain this improvement: only the processing, heavy-atom refinement and phasing differed. In fact, the quality of the AAS-improved SAD phases is comparable with that of conventional two-wavelength MAD phases. Thus, the gains in terms of phasing power that can be achieved by fully exploiting the AAS effects present in the data (*i.e.* without collecting new data) are comparable to those which in a conventional approach would require the collection of a complete second data set at a different wavelength. Although the conventional three-wavelength MAD phases are still superior to the AAS-improved SAD phases, it must be stressed that three times more data needed to be collected for the former. Also, it can be seen that even though very good data were obtained (three wavelengths, very high redundancy, no radiation damage), there is still a small improvement in the quality of phases that can be achieved by exploiting AAS (compare the phasing statistics for the three-wavelength MAD phasing with and without AAS).

## 9. Discussion

The previous examples show that AAS is likely to have an impact on most standard SAD or MAD experiments performed at an absorption edge. Indeed, AAS is intrinsic to the phenomenon of X-ray scattering and its occurrence is therefore not restricted to special types of experiments (*e.g.* those involving azimuthal scans). The question then arises as to why the effects of AAS have until now largely gone unnoticed in protein crystallography. This state of affairs is the consequence of a combination of several circumstances.

(i) In the earlier days of the development of the MAD method, many experiments were conducted on bending-magnet beamlines at second-generation synchrotrons. The relatively large divergence of the beam delivered by these sources often gave rise to monochromatic X-rays of rather poor spectral purity. In the study of Fanchon & Hendrickson (1990 ▶), the weakness of the observed AAS-induced intensity modulations was attributed to the rather large energy bandpass of the beamline used (Δ*E* ≃ 10 eV). The spectral purity of the incident X-ray beam is a critical quantity since a poor energy resolution can completely smear out the effects of AAS. With the advent of undulator-based beamlines at third-generation synchrotrons, X-ray beams of greater spectral purity are now delivered routinely and many experimentalists have made anecdotal observations of dichroism, *i.e.* variations in X-ray absorption spectra as a function of crystal orientation. Occasionally, such observations were incorrectly attributed to radiation damage or changes of the oxidation state of the anomalously scattering atoms in the crystal. For further discussion of these aspects, see Bricogne *et al.* (2005 ▶).
(ii) As we have shown here, in a standard rotation experiment (*i.e.* an experiment in which no special scans are performed), the main effect of AAS is the breaking of the equivalence of symmetry-related reflections. Furthermore, the potential phase information obtained by AAS is contained in the genuine intensity differences between these symmetry-related reflections. Alas, the widespread practice of merging data prior to phasing completely scrambles the effects of AAS. Although AAS is present in many unmerged data sets, the intensity differences between symmetry-related reflections are often relatively modest and of the same order of magnitude as Friedel differences. However, in comparison to Friedel differences, AAS gives rise to intensity differences between symmetry-related reflections that follow a more complex pattern. Thus, even if a data set is affected by AAS, this does not usually have a clearly discernible impact on the merging statistics and it can therefore remain hidden.
(iii) On protein crystallography beamlines, the single rotation axis has almost universally been oriented horizontally, *i.e.* exactly along the direction of polarization of the X-ray beam. Thus, as the crystal is rotated during data collection, the direction of X-ray polarization remains constant with respect to the orientation of the bonds around anomalously scattering atoms in the crystal. As we have shown here, if symmetry elements are closely aligned with the direction of X-ray polarization, the equivalence of certain groups of symmetry-related reflections is restored, even in the presence of AAS. In general, whenever the direction of polarization is aligned with the highest symmetry axis (the unique axis) of the crystal, it follows from the considerations discussed in §§[Sec sec5.3]5.3 and [Sec sec5.4]5.4 that the symmetry-breaking effects of AAS will be minimal (they will be zero in the forward-scattering approximation), except in cubic space groups. In orthorhombic space groups, the effects of AAS will be minimized whenever the rotation axis is aligned with any of the three twofold axes.
(iv) Even if residual effects of AAS remain present in a merged data set, these can be treated (although not actively exploited) as ‘anomalous non-isomorphism’ in the maximum-likelihood formalism of heavy-atom refinement and phasing (de La Fortelle & Bricogne, 1997 ▶).

The reasons listed above explain why AAS has so far remained a relatively invisible phenomenon in protein crystallography and also why it does not generally have a deleterious effect on the success of the SAD and MAD methods. On the other hand, we have shown here that if AAS is fully exploited, substantial gains in phase information can be obtained.

The prospect of fully exploiting the phase information generated through AAS brings into consideration entirely new strategies of data collection. Crystals could be intentionally misaligned in order to maximize the AAS-induced inequivalence between symmetry-related reflections. Furthermore, if the rotation axis is chosen along a direction that does not coincide with the direction of X-ray polarization (*e.g.* using a vertical scan axis) the effects of AAS in the data set will be significantly boosted since the **f**′ and **f**′′ tensors will then be sampled over a much wider range of polarization directions than is currently the case (Schiltz & Bricogne, work to be published). Indeed, on undulator-based beamlines at third-generation synchrotrons there are no compelling reasons to limit oneself to a horizontal rotation axis.

These new approaches will be greatly facilitated by the systematic deployment of standard or mini-kappa gonio­meters on synchrotron beamlines. Moreover, the future use of goniometers with a vertical spin axis, designed for the purposes of gaining mechanical stability in the handling of microcrystals, will result in the most general AAS effects being ubiquitous in data sets recorded on such instruments. The magnitude of the AAS effects themselves could be enhanced by reducing the bandwidth of the X-ray beam through the use of higher order reflections (*e.g.* 311) from Si crystals in monochromators. This would come at the expense of a reduced X-ray beam flux, but it would provide a more productive alternative for attenuating the incident beam at third-generation synchrotron beamlines than the widely used practice of using absorbers.

## 10. Conclusion

In retrospect, it appears that AAS, which is a well known physical complication of anomalous scattering at an absorption edge, was considered at some stage as a potential threat to the simplicity of the MAD method but was then ignored to all intents and purposes. We have shown in this work that if appropriate steps are taken to preserve the original measurements in which these effects appear, a suitable generalization of current phasing methods can accommodate them and deliver extra phasing power compared with conventional approaches using the same data. Examples were given that show improvements in the phases which are typically of the same order of magnitude as those obtained in a conventional approach by adding a second-wavelength data set to a SAD experiment. Thus, the exploitation of AAS can give access to a two-wavelength map quality with single-wavelength measurements. Such a gain is particularly significant, since radiation damage can frequently preclude the collection of a second-wavelength data set. It may thus also be worthwhile revisiting SAD or MAD data sets where the quality of the phases was too marginal to provide an interpretable map.

Now that we have established how to handle and exploit AAS effects, new possibilities are opened to use them deliberately by incorporating them in the design of experiments. We conclude by citing Templeton & Templeton (1982 ▶) who wrote that AAS

adds a new dimension of complexity to the theory of X-ray scattering. By introducing an error into the conventional methods of computation, it offers a handicap […] to exploit the maximum effects at the absorption edges for solving the phase problem. Thus from a pessimistic point of view it is a setback. We adopt the opposite view: where there is a complication there is the opportunity of sharper, more penetrating methods for extracting information from diffraction experiments.

## Figures and Tables

**Table 1:** Data-collection and processing statistics for the brominated Z-DNA duplex d(CGCG[BrU]G) Values in parentheses are for the outer resolution shell.

	Data set (I)	Data set (II)	Data set (III)
X-ray polarization direction† (**p**)	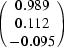	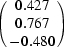	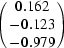
X-ray wavelength (Å)/photon energy (keV)	0.9199/13.477		
Rotation per image (°)	1	1	1
Exposure time per image (s)	2.2	2.2	2.2
Total No. of images	145	145	149
Space group	*P*2_1_2_1_2_1_		
Unit-cell parameters (Å, °)	*a* = 17.34, *b* = 32.07, *c* = 44.34, α = β = γ = 90		
Resolution limits (Å)	32.1–1.10 (1.16–1.10)	30.0–1.10 (1.16–1.10)	32.1–1.10 (1.16–1.10)
No. of measured reflections	41485 (1004)	41428 (1001)	42675 (1041)
No. of unique reflections in Laue group *mmm*	8303 (443)	9306 (603)	8542 (497)
No. of unique reflections in Laue group 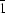	26133 (695)	26151 (694)	26371 (700)
*R*_meas_ in point group 222‡	0.073 (0.292)	0.090 (0.289)	0.059 (0.174)
*R*_meas0_ in Laue group *mmm*§	0.096 (0.302)	0.130 (0.321)	0.066 (0.252)
*R*_meas_ in point group 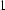	0.090 (0.288)	0.104 (0.401)	0.074 (0.253)
*R*_meas0_ in Laue group 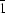	0.108 (0.305)	0.153 (0.328)	0.071 (0.257)

† All vectors are expressed on a crystal Cartesian basis (**e**_*x*_, **e**_*y*_, **e**_*z*_) which sets **e**_*x*_ parallel to **a** and **e**_*z*_ parallel to c*.

‡ Multiplicity-weighted merging *R* factor, keeping Bijvoet pairs separate (*i.e.* computed in the crystal point group).

§ Multiplicity-weighted merging *R* factor, not keeping Bijvoet pairs separate (*i.e.* computed in the crystal Laue group).

**Table 2:** Refined parameters of the two Br atoms in crystals of d(CGCG[BrU]G) Refinement was carried out in *SHARP* against unmerged data from all three crystal orientations. Crystal orientation data were used to compute the **p** and **p**′ vectors for each reflection record. The AAS properties of the Br atoms were modelled by second-rank symmetric **f**′′ tensors with refineable elements.

Parameters	Br 1	Br 2
*x*	0.0512 (2)	0.6016 (2)
*y*	0.3094 (1)	0.7257 (1)
*z*	0.7750 (1)	0.4925 (1)
*B* (Å^2^)	13.1 (2)	12.7 (2)
*B*_aniso_ (Å^2^)	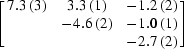	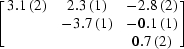
Occupancy	1 (not refined)	1 (not refined)
*f*′	−9.5 (not refined)	−9.5 (not refined)
**f**′′ tensor	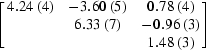	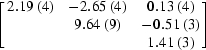
Eigenvalues of **f**′′	1.28, 1.55, 9.23	1.32, 1.40, 10.52
Eigenvector corresponding to the largest eigenvalue	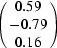	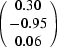
Direction of C—Br bond	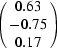	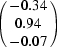

**Table 3:** Refined parameters of the Br atoms in crystals of d(CGCG[BrU]G) Refinement was carried out in *SHARP* against unmerged data from all three crystal orientations. Individual *f*′′ factors were refined for symmetry-related (*s* = 1…4) Br atoms in each of the three data sets (corresponding to three different crystal orientations).

Parameters	Br 1	Br 2
*x*	0.0513 (2)	0.6017 (2)
*y*	0.3094 (1)	0.7257 (1)
*z*	0.7749 (1)	0.4925 (1)
*B* (Å^2^)	13.1 (2)	12.6 (2)
*B*_aniso_ (Å^2^)	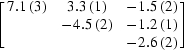	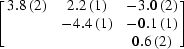
Occupancy	1 (not refined)	1 (not refined)
*f*′	−9.5 (not refined)	−9.5 (not refined)
Data set (I)		
*f*_*s*=1_′′	3.33 (6)	1.69 (5)
*f*_*s*=2_′′	3.56 (6)	1.77 (5)
*f*_*s*=3_′′	5.21 (7)	2.83 (6)
*f*_*s*=4_′′	4.84 (7)	2.76 (6)
Data set (II)		
*f*_*s*=1_′′	3.11 (7)	5.09 (7)
*f*_*s*=2_′′	2.41 (7)	4.52 (7)
*f*_*s*=3_′′	8.15 (9)	8.51 (9)
*f*_*s*=4_′′	7.68 (8)	7.68 (8)
Data set (III)		
*f*_*s*=1_′′	1.20 (5)	1.54 (5)
*f*_*s*=2_′′	2.34 (5)	1.79 (5)
*f*_*s*=3_′′	1.54 (5)	1.37 (5)
*f*_*s*=4_′′	1.60 (5)	1.56 (5)

**Table 4:** Data-collection and processing statistics for PPAT Values in parentheses are for the outer resolution shell.

X-ray wavelength (Å)/photon energy (keV)	0.97918/12.6636
Rotation per image (°)	0.5
Exposure time per image (s)	45
Total No. of images	480
Space group	*I*23
Unit-cell parameter (Å)	*a* = 136.23
Resolution limits (Å)	36.42–2.20 (2.32–2.20)
No. of measured reflections	599090 (89990)
No. of unique reflections in Laue group *m*3	21494 (3121)
No. of unique reflections in Laue group 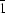	241760 (35446)
*R*_meas_ in point group 23†	0.113 (0.288)
*R*_meas0_ in Laue group *m*3‡	0.113 (0.288)
*R*_meas_ in point group 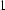	0.114 (0.280)
*R*_meas0_ in Laue group 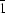	0.114 (0.280)

† Redundancy-independent (multiplicity-weighted) merging *R* factor, keeping Bijvoet pairs separate (*i.e.* computed in the crystal point group).

‡ Redundancy-independent (multiplicity-weighted) merging *R* factor, not keeping Bijvoet pairs separate (*i.e.* computed in the crystal Laue group).

**Table 5:** Refined parameters of the Se atoms in crystals of PPAT Refinement was carried out in *SHARP* against unmerged data and individual *f*_*j*,*s*_′′ factors were refined for symmetry-related (*s* = 1…12) Se atoms. Sites *s* = 1, 2, 3, 4 are related by twofold rotations about 〈100〉 and similarly for sites *s* = 5, 6, 7, 8 and for sites *s* = 9, 10, 11, 12. The set of sites *s* = 1, 2, 3, 4 is related to the set of sites *s* = 5, 6, 7, 8 and to the set of sites *s* = 9, 10, 11, 12 by threefold rotations about 〈111〉. Explicitly, the point operators are as follows: 
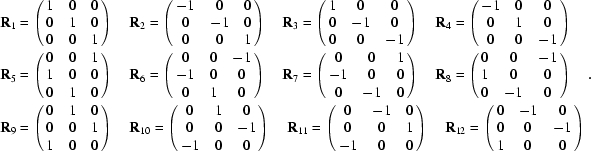
It can be seen that for atoms which are related by a twofold symmetry operation the *f*′′ parameters refine to similar values, whereas for atoms which are related by a threefold symmetry operation the refined *f*′′ values differ widely.

	*f*_*j*,*s*_′′ for symmetry-related sites											
Se site (*j*)	*s* = 1	2	3	4	5	6	7	8	9	10	11	12
A1	6.40	5.89	6.07	6.07	8.05	8.69	8.39	8.36	8.82	9.24	8.83	9.42
A2	3.73	3.89	4.21	3.84	7.64	7.72	8.10	7.98	8.63	9.01	9.10	8.97
A3	7.49	7.32	7.32	6.66	5.19	4.96	5.62	4.81	5.92	5.94	6.54	6.82
A4	5.41	5.62	5.54	5.04	3.74	3.82	4.34	4.20	5.16	5.57	5.89	5.64
A5	5.54	6.12	5.94	6.65	7.94	8.73	7.95	8.18	5.14	5.61	6.00	5.73
A6	9.85	9.52	10.10	9.61	9.23	9.04	9.77	9.67	4.14	3.90	4.55	4.92
A7	8.73	7.55	8.47	7.71	10.89	11.08	11.15	10.88	11.06	10.36	9.38	10.59
A8	6.08	5.93	6.50	6.18	6.33	6.36	6.43	6.90	5.95	5.78	6.31	6.35
B1	5.48	5.79	5.59	6.19	7.72	8.11	8.29	8.17	7.06	7.37	7.64	7.63
B2	5.01	5.18	4.69	5.77	9.31	10.66	9.05	9.99	8.02	8.17	8.42	8.25
B3	9.61	9.36	9.17	8.90	8.33	6.90	8.15	7.31	8.04	7.44	8.31	8.32
B4	6.91	6.10	5.78	6.26	7.14	6.23	7.13	6.63	5.26	5.32	5.81	5.42
B5	6.90	7.45	6.97	7.88	7.06	7.76	6.65	7.53	8.45	8.29	8.98	8.58
B6	9.34	9.39	9.07	8.95	3.48	3.19	3.74	3.31	8.74	9.06	9.28	9.22
B7	5.62	6.77	5.74	6.27	6.94	6.41	6.56	6.33	7.79	8.14	7.73	7.23
B8	7.82	8.46	8.58	8.76	7.54	7.89	7.63	8.08	6.88	6.54	7.51	7.32

## Footnotes

1 These relations are equivalent to equation (3) in Templeton & Templeton (1982 ▶). In Fanchon & Hendrickson (1990 ▶) they also appear in equation (3) and in Kirfel *et al.* (1991 ▶) they appear in equation (8). Both Templeton & Templeton (1982 ▶) and Kirfel *et al.* (1991 ▶) explicitly chose for the vectors **u**, **v**, **u**′ and **v**′ directions that are perpendicular and parallel, respectively, to the plane of diffraction. These directions are denoted *s*, *s*′, *p* and *p*′ by Templeton & Templeton (1982 ▶) or σ, σ′, π and π′ in Kirfel *et al.* (1991 ▶). However, the choice of these directions is completely free, as was noticed by Fanchon & Hendrickson (1990 ▶). Below, we show that a particular choice of vectors **u**, ** v**, **u**′ and **v**′ can considerably simplify the expressions in the case of linearly polarized X-rays.

2 This also explains why in macromolecular crystallography the effects of AAS are much more pronounced in nonforbidden reflections than in forbidden ones. In forbidden reflections, the normal part of the structure factor, *F*(**h**), is always zero, so that the AAS-induced intensity modulations only arise from the terms *G*_*p*′*p*_(**h**) and , which are always small. However, for nonforbidden reflections (22) can be explicitly written as *I*(**h**) ≅ . As a con­sequence of the quadrature relationship between intensities and structure factors, the modulations arising from *G*_*p*′*p*_(**h**) in the third term of the right-hand side of this equation are ‘boosted’ by the multiplication by the usually much larger |*F*(**h**)|.

3 We will henceforth use the term symmetry-related reflections rather than symmetry-equivalent reflections, since in the presence of AAS these reflections are no longer equivalent.

4 This operation is similar to the reflection of *F*(−**h**) through the real axis on the complex plane when building the Harker construction with Friedel pairs in the case of anomalous scattering (North, 1965 ▶). In that case, the Harker construction is actually set up with *F*(**h**) and , where  is the complex conjugate of *F*(−**h**).

5 It also holds exactly, at any scattering angle, for reflections **h** ⊥ **p**, *i.e.* for diffraction in the vertical plane. This was, for example, the case for all measurements that were carried out on four-circle diffractometers with a point detector by Templeton and Templeton.

6 To put it more succinctly: anomalous scattering is similar to X-ray absorption, but with phase shifts (Bricogne & Schiltz, 2000 ▶).
